# Is the Pass/Fail System Applicable to a Medical School in Korea?

**DOI:** 10.3352/jeehp.2007.4.3

**Published:** 2007-12-20

**Authors:** Mee Young Kim

**Affiliations:** Department of Medical Education, College of Medicine, Hallym University, Chuncheon, Korea.

**Keywords:** Medical Education, Education Evaluation, Pass/Fail System

## Abstract

To determine whether a pass/fail system is more appropriate for medical education instead of a grade-based system, a survey of medical students and faculty members of Hallym University, Korea, was taken. A questionnaire was delivered to 54 junior students and 36 faculty members from a medical school in Korea and analyzed. Of these participants, 37.7% of students and 36.1% of faculty agreed to the pass/fail system, while 28.3% of students and 52.8% of faculty objected to it. The most frequent reason for objection was the potential decrease in learning achievement. A pass/fail system should be considered after persuasion of the students and faculty to think positively of this system.

Traditionally, medical schools in Korea have used five-step letter grading systems, which have assessed class performance using A, B, C, D, or F. In some instances, some classes are evaluated by the pass/fail system (P/F system). However, some medical schools outside of Korea have adopted the numeric grading system (a continuous numeric system), letter grading system (A^+^, A, B^-^, etc.), honors/pass/fail system (whereby a certain percentage of students obtain the 'honors' grade), and P/F system.

Recently, teaching methods in medical schools have begun to move away from the didactic lecture, instead espousing team-learning and self-learning. To augment these new trends and to motivate active participation, reform of the evaluation system-eg, via the P/F system-was considered.

The P/F system has been reported to stimulate more vigorous study in students, to ease the anxiety caused by competition, and to motivate cooperative group work [1]. Also, in graduates of medical schools where the P/F system had been adopted, the clinical performance abilities of these students increased [2], and the solidarity of the student group was strengthened [3]. Based on previous reports, our survey attempted to gauge the opinion of medical students and faculty members on whether the P/F system would be applicable to medical school.

The questionnaire for this survey on the P/F system was delivered to 54 junior students (21 female, 28 male, 5 unidentified) and 36 faculty members (11 female, 25 male) in a medical school. The average duration of education of the faculty was 9.77±5.6 years. Initially, the agreement or objection to the system was noted, and potential effects of the P/F system were then presented. Open opinions were also gathered. The content of the questionnaire is described in Table 1. Unanswered ones were treated as missing values.

Of all survey participants, 37.7% of students and 36.1% of faculty agreed with the P/F system, while 28.3% of students and 52.8% of faculty objected to this system. Males (44.2%) agreed more than females (18.8%) (Table 2). Students expected more active participation in class without the stress of grades in this system. Half of the students and faculty agreed on the benefits of more active cooperative group work.

Half of all faculty, however, worried about the potential decrease in learning achievement. Furthermore, 79.6% of students and 66.7% of faculty worried about the difficulty in setting the cutoff score for a "pass" (Table 1).

In the open-ended questions, the reasons for agreement by students were the possibility of in-depth study and a cooperative environment among students without the worry over grades. Many faculty agreed, since the ultimate goal of medical education is the sufficient achievement of minimum requirements to be a doctor.

Reasons for objection by students and faculty were the lack of motivation to study and the likely decrease in learning achievement. Students had qualms over setting too high a cutoff score and the greater potential for failure in courses. The faculty were worried about the resistance students might generate if the cutoff score was so high that massive failure occurred. Also, faculty were afraid that the decision to award scholarships may be difficult if it was based solely on a binary pass/fail system.

Although the P/F system has some merits, there are still many obstacles to this system, particularly with regard to a potential decrease in learning achievement and a lack of motivation for study. Although reports on use of the P/F system in the United States have been positive, it is uncertain that these results can be recapitulated in Korea [1, 2].

In our survey, positive responses to P/F system were based on anticipated cooperative work. However, negative responses developed over worries that learning achievement will decline. These results suggest that while the study environment may be improved, the total time spent learning may be decreased. Also, students said that they could study harder if there was no stress related to high grade achievement, a rationale that the faculty did not believe.

Concerns about cutoff score determination by both students and faculty were substantial. A reasonable selection of the cutoff score should be considered, and should be reviewed to adapt the modified Angoff or Bookmark method for setting such scores, if necessary.

Meanwhile, compensation or motivation for high performance in students is another difficult task. The faculty mentioned new subject criteria for scholarship awards. A previous report suggested that if numeric grades were used for evaluation, learning achievement can be anticipated and much information can be deduced, a result that was not possible in the P/F system [4].

Program directors for recruitment of medical residents prefer the numeric grade system [5, 6]. Therefore, the experience in other medical schools should be reviewed precisely. It has been reported that the grading system should be more lucidly defined and specified in order to decrease grade inflation [7]. There are also reports concluding that it is better to categorize grades into four or five marks [8], that the letter grading system is highly reliable [9], and that the grading system is better than the P/F system with regard to acquisition of minimal competency requirements in bedside nursing education and compensation to high-performance students [10].

Still, there are many problems that need solved in implementing the P/F system. Team-learning or group study and self-directed study should be introduced in the premed period in order to provide a familiar environment for the P/F system. The setting of the cutoff score and a valid evaluation method should be prepared. The motivation of high-performance students should also be considered. Grading systems, such as the recruitment system of internship by a hospital, are sometimes necessary, and it will be challenging to report learning achievement in the P/F system in such a recruitment process.

Although there are some difficulties at hand, the P/F system evidently has some merit, such as the mitigation of stress, as well as the motivation for cooperative work. This system should be considered in medical schools in Korea after considerable support by students and faculty to think positively of this system and after providing a suitable environment in which to establish it [3].

## Figures and Tables

**Table 1:**
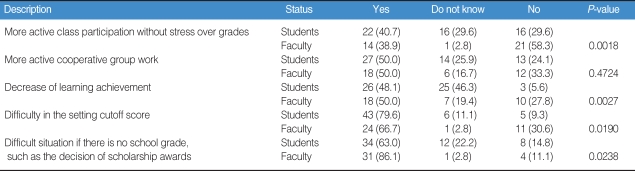
Descriptive opinions to the P/F system (%)

**Table 2:**
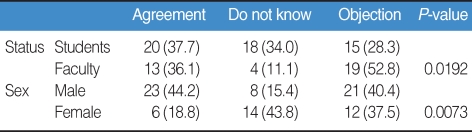
Agreement or objection to the P/F system (%)
